# Common molecular mechanism and immune infiltration patterns of thoracic and abdominal aortic aneurysms

**DOI:** 10.3389/fimmu.2022.1030976

**Published:** 2022-10-21

**Authors:** Bin He, Ya Zhan, Chunyu Cai, Dianyou Yu, Qinjiang Wei, Liping Quan, Da Huang, Yan Liu, Zhile Li, Li Liu, Xingshou Pan

**Affiliations:** ^1^ Graduate School of Youjiang Medical University for Nationalities, Baise, China; ^2^ The Third Hospital of MianYang, Sichuan Mental Health Center, MianYang, China; ^3^ Department of Cardiology, Affiliated Hospital of Youjiang Medical University for Nationalities, Baise, China; ^4^ College of Clinical Medicine, Youjiang Medical University for Nationalities, Baise, China

**Keywords:** aortic aneurysms, machine-learning, biomarkers, immune cell infiltration, whole exome sequencing

## Abstract

**Background:**

Aortic disease (aortic aneurysm (AA), dissection (AD)) is a serious threat to patient lives. Little is currently known about the molecular mechanisms and immune infiltration patterns underlying the development and progression of thoracic and abdominal aortic aneurysms (TAA and AAA), warranting further research.

**Methods:**

We downloaded AA (includes TAA and AAA) datasets from the GEO database. The potential biomarkers in TAA and AAA were identified using differential expression analysis and two machine-learning algorithms. The discrimination power of the potential biomarkers and their diagnostic accuracy was assessed in validation datasets using ROC curve analysis. Then, GSEA, KEGG, GO and DO analyses were conducted. Furthermore, two immuno-infiltration analysis algorithms were utilized to analyze the common immune infiltration patterns in TAA and AAA. Finally, a retrospective clinical study was performed on 78 patients with AD, and the serum from 6 patients was used for whole exome sequencing (WES).

**Results:**

The intersection of TAA and AAA datasets yielded 82 differentially expressed genes (DEGs). Subsequently, the biomarkers (*CX3CR1* and *HBB*) were acquired by screening using two machine-learning algorithms and ROC curve analysis. The functional analysis of DEGs showed significant enrichment in inflammation and regulation of angiogenic pathways. Immune cell infiltration analysis revealed that adaptive and innate immune responses were closely linked to AA progression. However, neither *CX3CR1* nor *HBB* was associated with B cell-mediated humoral immunity. *CX3CR1* expression was correlated with macrophages and *HBB* with eosinophils. Finally, our retrospective clinical study revealed a hyperinflammatory environment in aortic disease. The WES study identified disease biomarkers and gene variants, some of which may be druggable.

**Conclusion:**

The genes *CX3CR1* and *HBB* can be used as common biomarkers in TAA and AAA. Large numbers of innate and adaptive immune cells are infiltrated in AA and are closely linked to the development and progression of AA. Moreover, *CX3CR1* and *HBB* are highly correlated with the infiltration of immune cells and may be potential targets of immunotherapeutic drugs. Gene mutation research is a promising direction for the treatment of aortic disease.

## Introduction

Aortic aneurysms (AA) are the second most common aortic disease after atherosclerosis and can involve almost any part of the aorta. Older age, smoking, male gender and genetic susceptibility are strongly associated with the progression of AA. As the world’s population ages, the incidence of AA is increasing dramatically. Once an aortic aneurysm ruptures, it can be rapidly life-threatening, but patients are usually asymptomatic until the rupture event occurs (1, 2). Therefore, early diagnosis of AA and prevention of AA rupture are particularly important. The current treatment for AA includes both non-surgical and surgical treatment. Non-surgical treatment focuses on smoking cessation and blood pressure control. It has been established that the diameter, growth rate and symptoms of AA are important aspects in considering whether to operate. Surgical treatments include an open (AA resection and artificial vessel grafting) and an endovascular (endovascular abdominal aortic repair (EVAR) and less invasive endovascular stenting) approach (3). Over the years, these modalities have effectively reduced and prevented AA dilatation and rupture, which has saved the lives of many patients with AA. However, dilemmas are faced clinically, such as the lack of specific drugs targeting the pathogenesis of AA and the serious complications associated with surgical treatment (4). Thus, understanding the molecular mechanisms and immune pathways of AA can contribute to the development of drug targets and drug therapy for this deadly disease.

AA mainly includes two types in the thoracic or abdominal sections [thoracic and abdominal aortic aneurysms (TAA and AAA)]. The formation of AA is a complex and chronic process that results from the interplay of inherited and environmental factors. TAA and AAA are significantly different in terms of risk factors and pathophysiology. In this respect, current evidence suggests that the vascular smooth muscle cells (VSMCs) in the ascending aorta originate from the neural crest, whereas the abdominal aorta VSMCs originate from the endothelium and mesoderm (5, 6). Generally, TAA has a more solid genetic background than AAA since TAA can occur with Marfan and Loeys-Dietz syndrome due to autosomal gene mutation, while AAA is more associated with atherosclerosis (7). Despite these significant differences, they share many common features, such as a pathologically dilated aortic phenotype, loss of smooth muscle cells, inflammatory response, and altered extracellular matrix (8). However, whether TAA and AAA involve common molecular mechanisms such as immune infiltration during pathogenesis remains unclear. Indeed, understanding these mechanisms is critical for managing and treating AA. In this study, we innovatively combined TAA and AAA to explore potential key biomarkers or immune infiltration cells of AA progression compared to non-AA individuals by machine-learning and immuno-infiltration analysis algorithms.

In recent years, high-throughput sequencing and machine-learning algorithms have been widely applied in scientific research to identify novel genes associated with a variety of diseases, such as COVID19 (9), heart attack (10), atrial fibrillation (11) and cancer (12). These genes may serve as drug targets, disease diagnostic and prognostic biomarkers (13). In our study, we integrated two machine-learning algorithms (least absolute shrinkage and selection operator (LASSO) and support vector machine-recursive feature elimination (SVM-RFE)) to increase the accuracy of the signature genes for screening and further validated the diagnostic value of the identified biomarkers using receiver-operating characteristic (ROC) curve analysis. Moreover, we utilized two cutting-edge immune infiltration analysis algorithms, “CIBERSORT” and “ssGSEA” to deepen our understanding of the level of immune infiltration in TAA and AAA. In addition, the correlation between biomarkers and infiltrating immune cells was assessed using spearman’s rank correlation test. Overall, we identified biomarkers associated with the pathogenesis of immune infiltration in AA and provided the foothold for further research on drugs targeting characteristic molecules and immune cells.

Finally, we retrospectively assessed serum inflammation biomarkers and lipid levels in patients with AD, which revealed activation of the inflammatory milieu in aortic disease. The WES study provided further insight into gene mutations and whether biomarkers are abnormally mutated in aortic disease (14). Importantly, the sequencing analysis allowed the prediction of druggable variants of genes, which may lead to breakthroughs in treating aortic diseases.

## Materials and methods

### Data download and introduction

The microarray expression datasets (GSE47472, GSE57691 and GSE26155) related to AAA and TAA were downloaded from the Gene Expression Omnibus (GEO) database (http://www.ncbi.nlm.nih.gov/geo/) (15). The GSE57691 (16) and GSE26155 (17) datasets of AAA and TAA were used for the training group, and dataset GSE47472 (18) of AAA was used for the validation group. The training dataset of GSE57691 contained 49 cases of AAA, and 10 cases of controls, based on the GPL10058 platform. In addition, dataset GSE26155, based on the GPL5175 platform, contained 43 cases of TAA and 13 cases of controls. The validation dataset of GSE47472 contained 14 cases of AAA, and 8 cases of controls. The 92 AA (Treat, including 43 TAA and 49 AAA) samples belonging to the training group and the 14 AAA samples belonging to the validation group were derived from aortic wall tissue biopsy specimens, while the 23 controls (Con, including 13 TAA and 10 AAA) belonging to the training group and the 8 controls belonging to the validation group were derived from normal aortic tissue of organ donors. In the training dataset, samples that did not meet the diagnostic criteria for AAA and TAA were removed (3). Specifically, we removed the sample of 9 patients with aortic occlusive disease from the GSE57691 dataset and 30 patients with non-dilated aorta diameter (<40 mm) and 10 patients with aortic dilatation at borderline from the GSE26155 dataset. The mean maximum aorta diameter of AA and characteristics of the three datasets are presented in **Table 1**.

**Table 1:** Characteristics of the three AA datasets.

Datasets	Disease Type/groups	Contains	mean maximum aorta diameter, mm
GSE47472	AAA/validation	14 AAA 8 controls	62.6 ± 18.0 mm
GSE57691	AAA/training	49 AAA (29 large, 20 small) 10 controls	68.4 ± 14.3 (large)mm 54.3 ± 2.3 (small) mm
GSE26155	TAA/training	43 TAA 13 controls	53.6 ± 7.5mm

### Data merging, preprocessing, and screening of DEGs

The “sva” and “limma” R software packages (version 4.2.0) were used to merge, probe-annotate, normalize and batch-correct the data from GSE57691 and GSE26155 datasets (19, 20). Platform annotation files were utilized to convert probes in each dataset into gene symbols. The “combat” function of the “SVA” package was utilized to eliminate batch effects between the two datasets (21). Probes with the same gene symbol were averaged to define the gene expression for a given sample. Subsequently, the DEGs were identified based on the merged and preprocessed data files. The “pheatmap” package and “ggplot2” package were deployed to create DEGs heatmaps and volcano plots, respectively (22). The thresholds for DEGs included a log2 fold change (FC) > 1 and adjusted P-value < 0.05.

### Functional enrichment analysis

Gene Ontology (GO), Kyoto Encyclopedia of Genes and Genomes (KEGG) and Disease Ontology (DO) enrichment analyses were conducted on DEGs using the “clusterProfiler”, “enrichplot” and “DOSE” packages of the R software (23). “c2.cp.kegg.v7.4.symbols.gmt” and “c5.go.v7.4.symbols.gmt” obtained from the Molecular Signature Database (GSEA | MSigDB (gsea-msigdb.org)) were used for Gene Set Enrichment (GSEA) (24). The top five significantly enriched pathways gene sets were displayed. Adjusted P-values < 0.05 were statistically significant.

### Identification and verification of potential biomarkers in AAA and TAA

To identify potential biomarkers for AAA and TAA, the two machine-learning algorithms (“LASSO” and “SVM-RFE”) were applied to the DEGs of the training group. The “LASSO” algorithm was performed based on the R package “glmnet” and could identify genes significantly associated with AA and non-AA using ten-fold cross-validation (25). The “SVM-RFE” algorithm was performed based on the R package “e1071” to identify genes with a significantly strong distinguishing power (26). The genes obtained by these machine-learning methods were intersected. Subsequently, in the validation dataset of AAA, the expression levels of the overlapping genes were compared between the AA and control groups using a boxplot. The accuracy of the intersected genes as potential biomarkers for AA and control groups was assessed using ROC curve analysis.

### Immune infiltration analysis and potential biomarkers correlation with infiltrating immune cells

The R software’s “CIBERSORT” package was used to assess the level of immune cell infiltration based on 22 immune cell types of the “LM22” document (https://cibersort.stanford.edu/index.php) (27), The results were filtered using the screening criteria: P value< 0.05. The “ssGSEA” algorithm was used to assess the correlation of all gene expression profiles with the 28 immune cell types of “immune.gmt” based on R software’s “GSVA” packages (28). Depending on the results obtained by these two immuno-infiltration assays, the differential expression levels of 22 and 28 immune infiltrating cell types in the AA and non-AA were visualized using heatmaps and violin plots. The correlation analysis of 22 infiltrating immune cell types was visualized by the R software’s “corrplot” package. The degree of association between the 22 immune cell types and potential biomarkers was evaluated by “Spearman” correlation and visualized using R software’s “ggplot2” package.

### A retrospective clinical study of AD patients

Blood samples were collected from 78 patients with aortic dissection (AD) who were hospitalized at the Affiliated Hospital of Youjiang Medical University for Nationalities from 2007-2019. We retrospectively studied patient clinical information and serum inflammation markers and lipid levels. Serum inflammatory markers and lipid levels are tested using the Sysmex XN-1000™ Hematology Analyzer and Roche Cobas C702 fully automated biochemistry analyzer. The aortic computed tomography angiography (CTA) results of all included cases met the diagnostic criteria for AD (29). All patients provided written informed consent. The baseline characteristics of the patients and information after grouping according to Stanford classification were presented in **Table 2**. The Stanford classification divides dissections by the most proximal involvement into types A and B. The DeBakey classification divides AD into types I, II, and III based on the location of the primary rupture and the extent of entrapment. Our study was approved by the Ethics Committee of Affiliated Hospital of Youjiang Medical University for Nationalities (YYFY-LL-2016-06) and was in accordance with the principles of the Declaration of Helsinki.

**Table 2:** Baseline characteristics table of AD based on the Stanford classification.

Features/Groups	Participants (%) N=78	Stanford A (%) N=30	Stanford B (%) N=48	p-value
Gender:				0.499
Female	14 (17.9%)	7 (23.3%)	7 (14.6%)	
Male	64 (82.1%)	23 (76.7%)	41 (85.4%)	
Age(years)	54.5 ± 14.8	55.7 ± 17.0	53.7 ± 13.4	0.578
Ethnicity:				0.350
Buyei	4 (5.13%)	3 (10.0%)	1 (2.08%)	
Han	18 (23.1%)	8 (26.7%)	10 (20.8%)	
Yao	1 (1.28%)	0 (0.00%)	1 (2.08%)	
Zhuang	55 (70.5%)	19 (63.3%)	36 (75.0%)	
Smoking	34 (43.6%)	13 (43.3%)	21 (43.8%)	1.000
Hypertension	66 (84.6%)	20 (66.7%)	46 (95.8%)	0.001
Debakey:				<0.001
I	26 (33.3%)	26 (86.7%)	0 (0.00%)	
II	4 (5.13%)	4 (13.3%)	0 (0.00%)	
III	48 (61.5%)	0 (0.00%)	48 (100%)	

The Stanford A involves any part of the aorta proximal to the origin of the left subclavian artery (A affects ascending aorta). The Stanford B arises distal to the left subclavian artery origin.

### Whole Exome Sequencing

The peripheral blood samples of six patients with AD at the Affiliated Hospital of Youjiang Medical University for Nationalities were collected and underwent WES sequencing by Wuhan Huada Medical Laboratory Co. The six AD patients included five male patients and one female patient, two of whom (one male and one female) had lesions involving the thorax and abdomen, while the other four patients had lesions involving the thorax only. Subsequently, the Genome Reference Consortium Human Build 37 (GRCh37/hg19) was used to annotate the WES sequencing data. The mutation annotation results were further filtered using the following filters: ExAC_ALL, ESP6500, 1000G_EAS mutation frequency < 0.01 and VAF threshold > 0.05. The filtered mutation data were converted to the Mutation Annotation Format (maf) and further visualized using the “maftools” package in R (30). Finally, the Drug Gene Interaction Database (DGIdb) was applied to make predictions of potentially druggable genes based on the “drugInteractions” function of “maftools”.

## Results

### Data curation and DEGs screening in AAA and TAA

**Figure 1:**
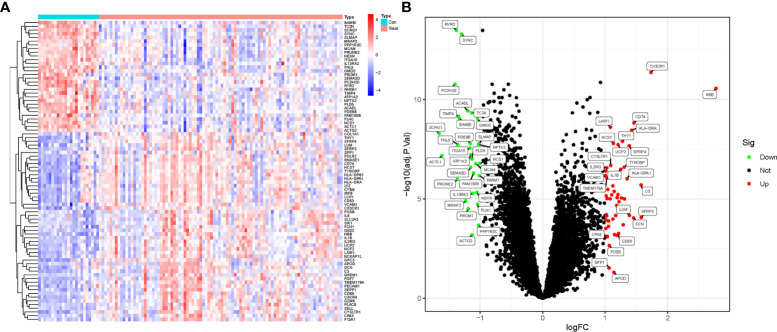
DEGs screening in AAA and TAA. **(A)** Heatmap of DEGs expression in aortic aneurysm (Treat) and non-aortic aneurysm (Con) groups. **(B)** Volcano plots of DEGs expression in Treat and Con groups.

Based on the research design, we downloaded and organized the list of gene symbols matrix information of training (GSE57691 and GSE26155) and validation (GSE47472) datasets (**Supplementary File 1**). Then, the training data from GSE57691 and GSE26155 datasets were merged, intersected, normalized and batch-corrected (**Supplementary File 2**). Based on the filtering criteria (log2 fold change (FC) > 1 and adjusted P-value < 0.05) for significant DEGs, a total of 82 DEGs were obtained. DEGs expression in the samples was visualized in a heatmap and volcano plot (**Figures 1A, B**). Collated results for all genes and DEGs are provided in **Supplementary File 3**.

### Functional correlation analysis

**Figure 2:**
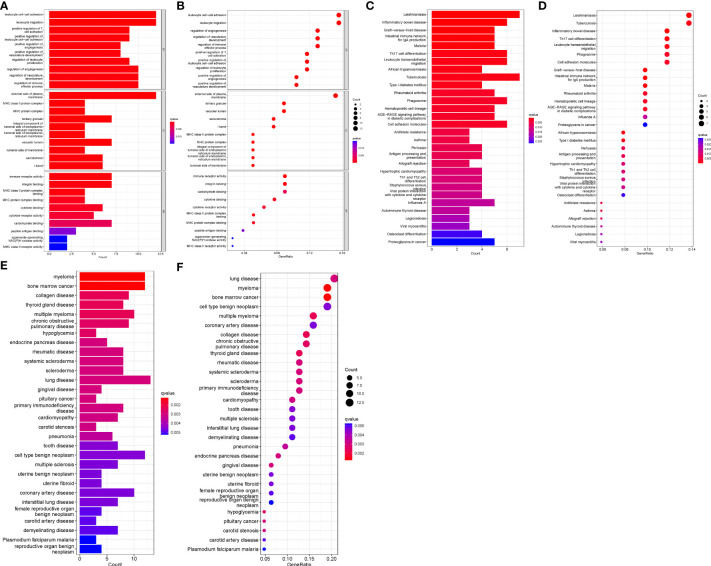
GO, KEGG and DO enrichment analyses. **(A)** Histogram of GO analysis. Enrichment significance increases with red color intensity. **(B)** Bubble plot representing GO analysis. Bubble size is proportional to the number of enriched genes. Red bubble color intensity increases with enrichment significance. **(C)** Histogram of KEGG analysis. **(D)** Bubble diagram representing KEGG analysis. **(E)** Histogram of DO analysis. **(F)** Bubble plot representing DO analysis.

GO, KEGG and DO enrichment analyses were conducted on DEGs to understand biological functions, signaling pathways, and disease mechanisms in AAA and TAA. Based on the screening criterion of adjusted P-value < 0.05, we obtained 606, 42 and 214 terms for the GO, KEGG and DO enrichment analyses, respectively (**Supplementary Files 4**–**6**). The top ten GO terms associated with biological process (BP), cellular components (CC) and molecular function (MF)) are shown in **Figures 2A, B**. The KEGG and DO analyses of the top 30 terms are displayed in **Figures 2C–F**. Significantly enriched BP terms included leukocyte cell-cell adhesion, leukocyte migration, regulation of angiogenesis, regulation of vasculature development and regulation of immune effector process. Significantly enriched CC and MF GO terms included external side of the plasma membrane, tertiary granule, vacuolar lumen, immune receptor activity and integrin binding. KEGG analysis showed significant enrichment in Leishmaniasis, Tuberculosis, Th17 cell differentiation, Leukocyte transendothelial migration and cell adhesion molecules. DO analysis showed that lung disease, coronary artery disease, myeloma and bone marrow cancer were highly associated with the DEGs.

### GSEA analysis

**Figure 3:**
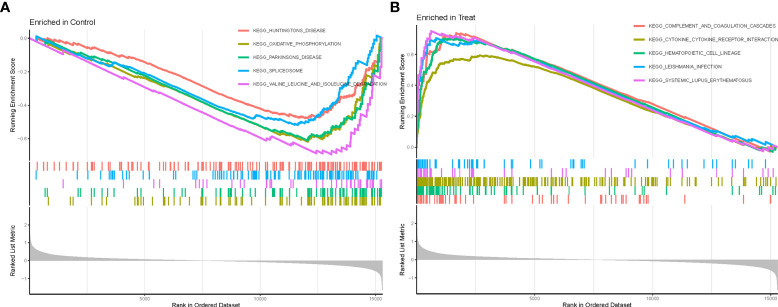
GSEA enrichment analysis. **(A)** KEGG pathway set scores enriched in the control group. **(B)** KEGG pathway set scores enriched in the Treat group.

We further performed GSEA enrichment analysis on all DEGs to better understand their potential functions and signaling pathways in AA (Treat) and non-AA (Con) cases (**Supplementary File 7**). Huntington’s disease and oxidative phosphorylation were significantly enriched in the Con group (**Figure 3A**). In contrast, the complement and coagulation cascades, cytokine-cytokine receptor interaction, hematopoietic cell lineage, Leishmania infections, and systemic lupus erythematosus were significantly enriched in the Treat group (**Figure 3B**).

### Identification of potential biomarkers in AAA and TAA by two machine-learning algorithms

**Figure 4:**
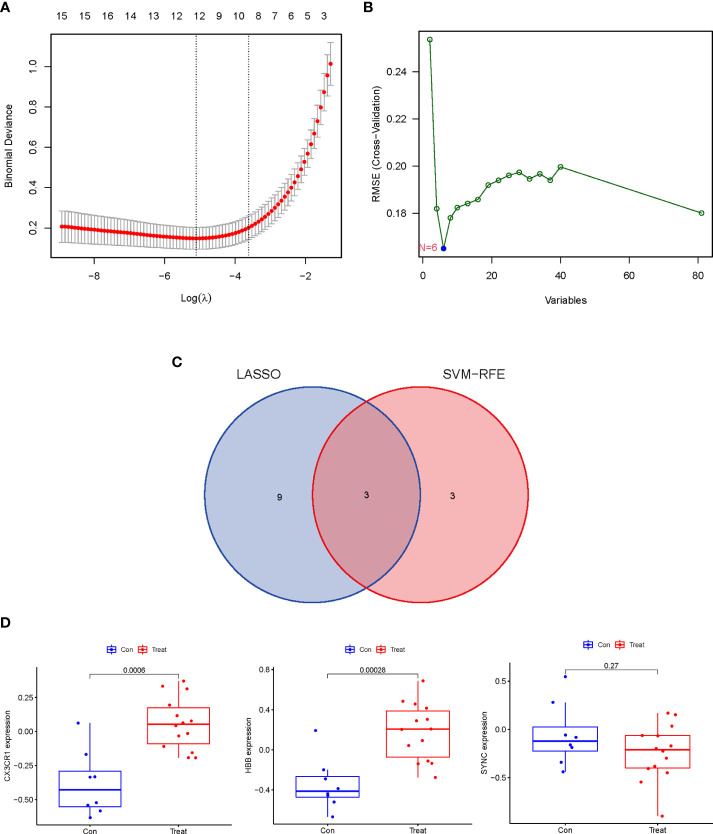
Identification and Verification of the discriminating power of potential biomarkers in AA. **(A)** Tuning feature selection in the “LASSO” model. The DEGs evaluated by 10-fold cross-validation in the “LASSO” regression model yielded 12 potential biomarkers. **(B)** The biomarkers screened by the SVM-RFE algorithm yielded 6 potential biomarkers. **(C)** Venn plot of potential biomarkers identified by “LASSO” and “SVM-RFE” algorithms. **(D)** The discrimination power of 3 potential biomarkers was verified in the validation dataset (GSE47472).

To identify common potential biomarkers in AAA and TAA, the DEGs obtained above were further screened using two machine-learning (“LASSO” and “SVM-RFE”) algorithms. 12 genes were screened by “LASSO” and 6 genes were screened by “SVM-RFE” algorithms (**Figures 4A, B**). The intersection of the results of the two algorithms yielded three genes (*SYNC*, *CX3CR1* and *HBB*) (**Figure 4C**).

### Verification of the discrimination power of the potential biomarkers and their diagnostic accuracy

**Figure 5:**
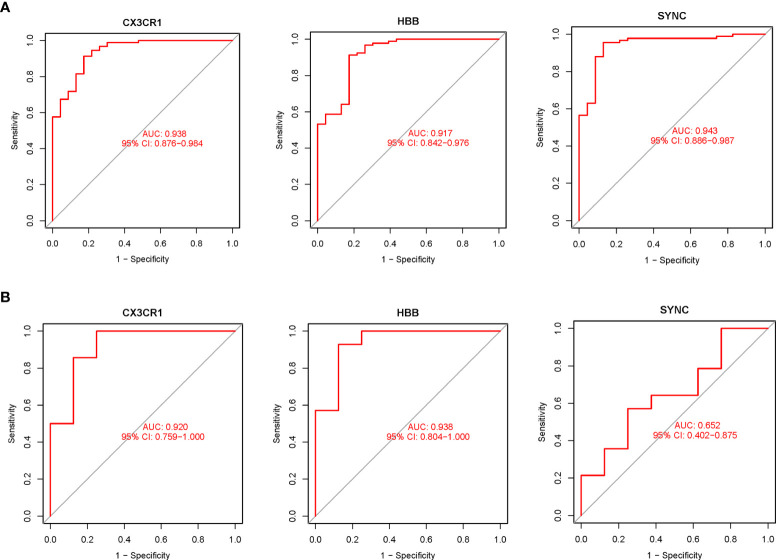
The diagnostic accuracy of the 3 potential biomarkers was assessed using ROC curve analysis. **(A)** Diagnostic ability of potential biomarkers in the training datasets. The AUC represents the diagnostic ability in Treat and Con groups. **(B)** Diagnostic accuracy of potential biomarkers in the validation datasets. The AUC represents the diagnostic accuracy in Treat and Con groups.

The expression levels of the three potential biomarkers between AA (Treat) and non-AA (Con) in the validation dataset GSE47472 were visualized in boxplots (**Figure 4D**). The expression levels of *CX3CR1* and *HBB* were significantly higher in the Treat group than in the Con group (P < 0.001). In contrast, *SYNC* did not differ between the two groups with a P value of 0.27. The diagnostic accuracy of the three potential biomarkers between the Treat and Con groups was assessed by ROC curve analysis in the training and validation datasets. In the training datasets, the AUC values of *CX3CR1*, *HBB*, and *SYNC* genes were 0.938, 0.917 and 0.943, respectively (**Figure 5A**). In the validation datasets, the AUC values of *CX3CR1*, *HBB* and *SYNC* were 0.920, 0.938, and 0.652, respectively (**Figure 5B**). These results suggest that *CX3CR1* and *HBB* have higher discrimination power and diagnostic accuracy and can be used as common biomarkers for AAA and TAA.

### Immune infiltration analysis and the correlation between infiltrating immune cell types

**Figure 6:**
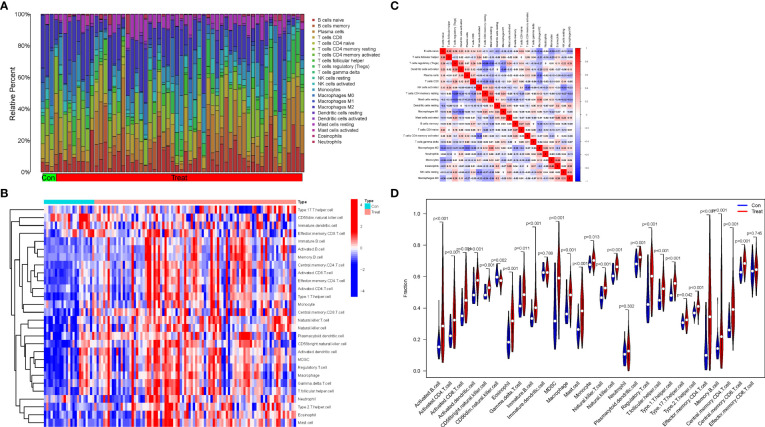
Immune infiltration analysis and the correlations between the infiltrating immune cell types. **(A)** The proportion of 22 infiltrating immune cell types in Treat and Con groups. **(B)** The distribution of the 28 infiltrating immune cell types in Treat and Con groups. **(C)** The correlations heatmap between the infiltrating immune cell types. **(D)** Violin plot demonstrating the differences between the 28 immune cell types in the Treat and Con groups.

Based on the normalized and merged gene expression data (**Supplementary File 2**), we compared Treat and Con groups by imputing the composition of immune cell populations using two algorithms, “CIBERSORT” and “ssGSEA” (**Figures 6A, B**; **Supplementary Files 8**, **9**). The correlations between the infiltrating immune cell types of “CIBERSORT” are shown in **Figure 6C**. Finally, a violin plot was generated to visualize the differences in immune infiltrating cell types of “ssGSEA” between the Treat and Con groups (**Figure 6D**, **Supplementary File 10**). The results showed a positive correlation between resting mast cells and resting memory CD4 T cells (r=0.50), while a negative correlation was found between regulatory T cells (Tregs) and resting memory CD4 T cells and resting mast cells (r=-0.59 and -0.53). In addition, both adaptive (activated CD4+ T cells, CD8+ T cells, activated B cell, effector memory CD4 + T cells and so on) and innate immune cells (activated dendritic cell, MDSC, natural killer cell and so on) were significantly higher in the Treat group than in the Con group. In contrast, infiltrations of neutrophils, effector memory CD8+ T cells and immature dendritic cells did not differ between the two groups.

### Correlation analysis between biomarkers (*CX3CR1* and *HBB*) and infiltrating immune cell types

**Figure 7:**
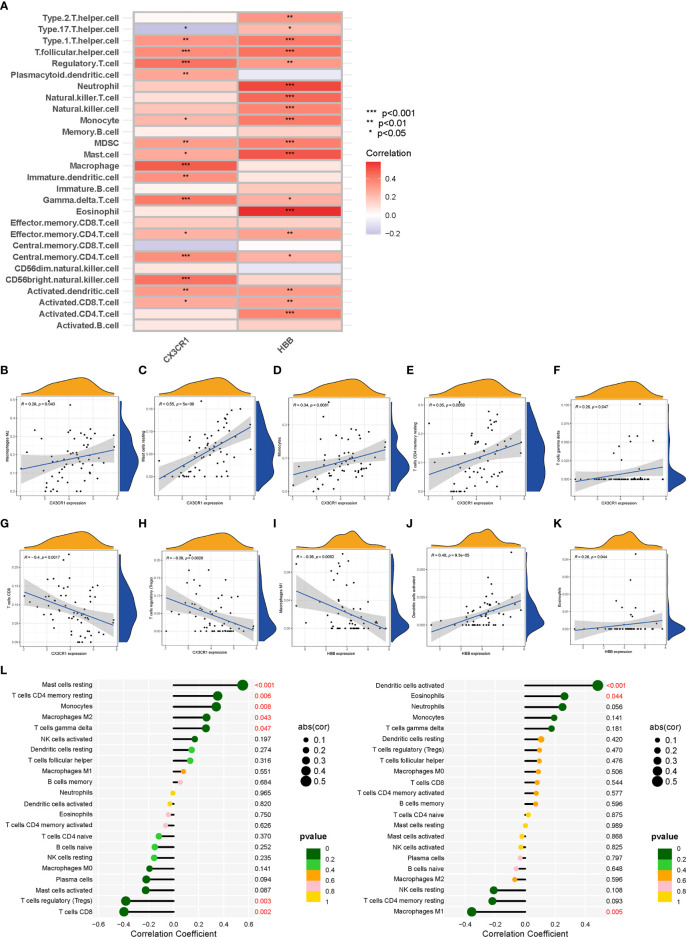
Correlation analysis between biomarkers (*CX3CR1* and *HBB*) and infiltrating immune cell types. **(A)** The relationship between 28 infiltrating immune cell types and two biomarkers; the redder the color, the more significant the difference. “***”, “**”, “*” represent P< (0.001, 0.01, 0.05). **(B–H)** Correlation between *CX3CR1* expression and infiltrating immune cell types. **(I–K)** Correlation between *HBB* expression and infiltrating immune cell types. **(L)** Correlation between *CX3CR1* and *HBB* and infiltrating immune cell types. The larger the dot, the stronger the correlation(cor). Numbers with P-value < 0.05 are marked red.

The correlations between the biomarkers *CX3CR1* and *HBB* and infiltrating immune cell types were assessed based on the results of “ssGSEA” (**Figure 7A**) and “CIBERSORT” analysis (**Figures 7B–L**). The correlation results of ssGSEA showed that macrophage (p<0.001), mast cell (p<0.05), MDSC (p<0.01), dendritic cell (p<0.01), monocytes (p<0.05), Tregs (p<0.001), CD56bright natural killer cell (p<0.001), central memory CD4 T cell (p<0.001), activated CD8 T cell (p<0.05), gamma delta T cell (p<0.001), T follicular helper cells (p<0.001), and type 1 T helper cell (p<0.01) were highly correlated with *CX3CR1*. In contrast, eosinophil (p<0.001), mast cell (p<0.001), neutrophil (p<0.001), dendritic cell (p<0.01), monocyte (p<0.001), Treg (p<0.01), natural killer cell (p<0.001), MDSC (p<0.001), activated CD4 T cell (p<0.01), activated CD8 T cell (p<0.01), effector memory CD4 T cell (p<0.01) and T helper cell (p<0.001) were highly correlated with *HBB*. “CIBERSORT” analysis showed that *CX3CR1* was positively correlated with resting mast cells (p<0.001), resting memory CD4 T cells (p<0.006), monocytes (p<0.008), M2 macrophages (p<0.043) and gamma delta T cells (p<0.047), and negatively correlated with CD8T cells (p<0.002) and Tregs (p<0.003) (**Supplementary File 11**). *HBB* was positively correlated with activated dendritic cells (p<0.001) and eosinophils (p<0.044) and negatively with M1 macrophages (p<0.005) (**Supplementary File 12**).

### Baseline characteristics and serum markers analysis in patients with AD

**Figure 8:**
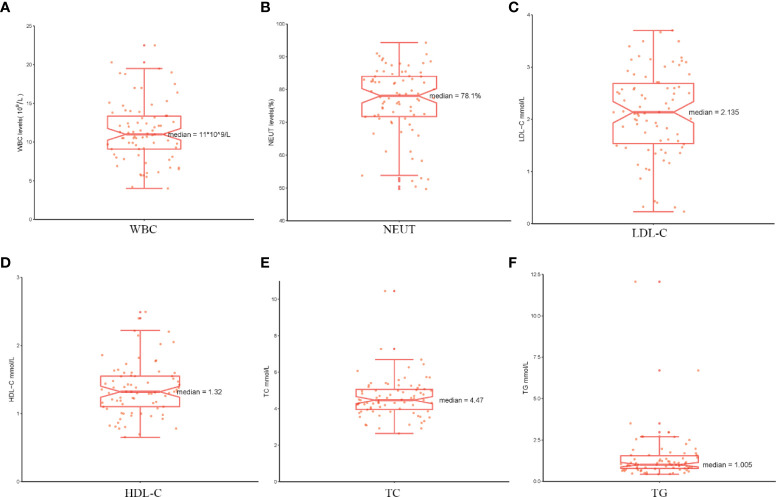
The serum markers analysis in patients with AD. **(A–F)** The serum levels of WBC, NEUT%, LDL-C, HDL-C, TC and TG are shown separately using box plots.

Analysis of the baseline patient characteristics (**Table 2**) showed that the prevalence was 82.1% and 17.9% in males and females, respectively, and the age of onset was 54.5 ± 14.8 years. 43.6% (n=34) of patients had a previous smoking history. 66.49% (n=66) of patients had a previous history of hypertension, of whom 95.8% (n=46) were Stanford type B patients (P < 0.001). Subsequently, we analyzed the statistics of serum inflammation and lipid levels in 78 patients with AD in **Table 3** and visualized them by box plots (**Figures 8A–F**). The median of white blood cell count (WBC) and neutrophil ratio (NEUT%) values in patients with AD were 11.0×10^9^/L and 78.1%, respectively. 63% (n=49) and 79% (n=62) of cases had WBC and NEUT% values greater than the upper limit of normal. The median of the four lipid indicators LDL-C, HDL-C, TC and TG, were 2.13 mmol/L, 1.32 mmol/L, 4.47 mmol/L, and 1.00 mmol/L, with 6% (n=5), 9% (n=7), 6% (n=5) and 18% (n=14) of cases with values above the upper limit of normal, respectively.

**Table 3:** The serum inflammatory and lipid levels in patients with AD.

Serum markers	Participants n=78M[IQR]	Conditions	Number n=78 (%)
WBC (10^9^/L)	11.0 [9.10;13.4]	>=10.0 *10^9^/L	49 (63%)
		<10.0 *10^9^/L	29 (37%)
NEUT (%)	78.1 [71.8;84.0]	>=70%	62 (79%)
		<70%	16 (21%)
LDL-C (mmol/L)	2.13 [1.54;2.69]	>=3.36mmol/L	5 (6%)
		<3.36 mmol/L	73 (94%)
HDL-C (mmol/L)	1.32 [1.10;1.55]	>=2.19 mmol/L	7 (9%)
		<2.19 mmol/L	71 (91%)
TC (mmol/L)	4.47 [3.96;5.06]	>=6.2 mmol/L	5 (6%)
		<6.2 mmol/L	73 (94%)
TG (mmol/L)	1.005 [0.78;1.54]	>=1.81 mmol/L	14 (18%)
		<1.81 mmol/L	64 (82%)

The serum inflammatory indicators include: white blood cell count (WBC) and neutrophil ratio (NEUT%); the reference values are: WBC (4.0~10) *10^9^/L and NEUT% (50%~70%), respectively. The lipid panel includes: Low-density lipoprotein cholesterol (LDL-C), High-density lipoprotein cholesterol (HDL-C), Total cholesterol (TC), and Triglycerides (TG); the reference values are: LDL -C (0~3.36mmol/L), HDL-C (0.9~2.19 mmol/L), TC (3.1~6.2 mmol/L), and TG (0.30~1.81 mmol/L).M(Median), IQR (InterQuartile Range).

### Whole exome sequencinganalysis

**Figure 9:**
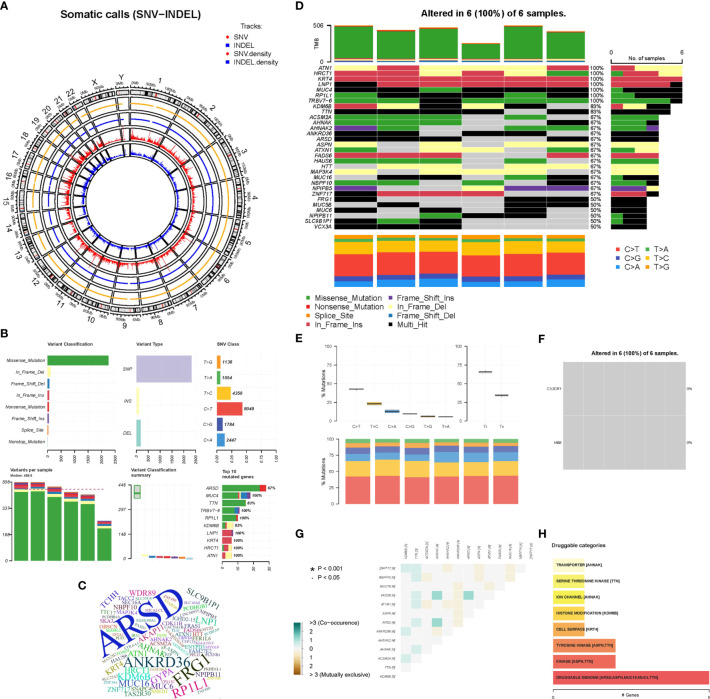
Whole Exome Sequencing analysis. **(A)** The circos plot of SNV-INDEL, the two outer tracks show the position of SNV and INDEL on the chromosome, and the two inner tracks show the distribution density. **(B)** The overall characteristics of the filtered mutation data. **(C)** The word cloud of the top 100 genes, the larger the font, the higher the variation frequency. **(D)** The oncoplot belongs to the top mutated 30 genes in each sample, different colors represent different variant classifications. **(E)** The overall distribution of the six different Ti/Tv in six samples. **(F)** The mutations of biomarkers (*CX3CR1* and *HBB*) in six samples. **(G)** Relevance Heatmap of the top 13 mutated genes. **(H)** The bar chart of potentially druggable variants genes.

As a preliminary investigation of genetic variation in aortic disease, peripheral blood samples from 6 AD patients were subjected to whole exome sequencing. The distribution density of unfiltered single-nucleotide variant (SNV) and INDEL in chromosomes following WES analysis is shown in **Figure 9A**. The overall characteristics of the filtered mutation data are shown in **Figure 9B**. The top 100 high-frequency mutation genes are shown in **Figure 9C**. The top 30 high-frequency mutation genes and their corresponding mutation types are shown in **Figure 9D**. Transitions (Ti), Transversions (Tv) and the overall distribution of the six different Ti/Tv are shown in **Figure 9E**. The mutations in biomarkers (*CX3CR1* and *HBB*) in the 6 samples are shown in **Figure 9F**. Correlation analysis of the top 13 mutated genes is shown in **Figure 9G**. Potentially druggable genes are shown in **Figure 9H**. It could be found that missense mutations accounted for the major part; the frequency of SNP was higher than that of insertions or deletions; C>T was the most common mutation in SNV, with a mutation frequency of about 40%, followed by T>C, with a mutation frequency of about 25%. The ratio of Ti to Tv in the six sequenced samples was approximately 2:1, and the median variance per sample was 495.5. Among the top mutated genes, 100% mutation frequency was observed for *ATN1*, *HRCT1*, *KRT4*, *LNP1*, *MUC4*, *RP1L1* and *TRBV7-6* and 83% for *TTN* and *KDM6B*. Insertion frameshift mutation, nonsense mutation and missense mutation were the main types of forward mutations. In contrast, the biomarkers *CX3CR1* and *HBB* were not found to have abnormal mutations in the six sequenced samples. In addition, the correlation analysis of 13 mutated genes in six samples showed no significant correlation. Finally, the potentially druggable genes in the Drug-Gene Interaction Database (DGIdb) included *ARSD, ASPN, MUC16, MUC4, and TTN*.

## Discussion

The development of high-throughput sequencing technology has brought medical research into the era of big data, while bioinformatics and artificial intelligence in medicine has enabled researchers to better analyze large amounts of sequencing data (31, 32). The combination of advanced machine-learning algorithms and medical research has led to tremendous advances in the pathogenesis, diagnosis, prognosis and treatment of various diseases (33). In this study, we innovatively merged TAA and AAA data and identified three potential biomarkers by two machine-learning algorithms (“LASSO” and “SVM-RFE”). Subsequently, we validated the discrimination power and diagnostic accuracy of the potential biomarkers in a validation dataset using ROC curve analysis. *CX3CR1* and *HBB* were finally identified as accurate and reliable biomarkers of AA. These biomarkers provide novel insights into the molecular mechanisms underlying the development and progression of TAA and AAA. However, in this study we only compared biomarkers in the AA and non-AA groups. In the future, more researchers and scientists may be able to compare different sexes, different sizes of mean maximum aorta diameter and different age groupings, and thus the results obtained will further deepen the understanding of the disease at different levels.

The pathology of AA is thought to involve localized chronic inflammatory response with persistent angiogenesis and imbalance in extracellular matrix protein hydrolysis, leading to progressive weakening and dilatation of the aortic wall (34, 35). During chronic vascular inflammation, chemokines play a crucial role by mediating the activation of inflammatory and immune cells and their aggregation to the vessel wall (36, 37). Current evidence suggests that *CX3CL1* (also known as fractalkine), a specific member of the chemokine family, can act as an adhesion molecule through a membrane-bound form and as a chemoattractant in vascular inflammatory processes through a soluble form (38, 39). Interestingly, it has been shown that *CX3CR1* is a receptor for *CX3CL1* and is expressed on the surface of a variety of innate and adaptive immune cells, such as T lymphocytes, monocytes, natural killer cells, mast cells, platelets, and vascular smooth muscle cells (40–43). Moreover, *CX3CL1* on cell surfaces can play an important role in inflammatory vascular diseases by promoting migration, adhesion and proliferation of these immune cells expressing *CX3CR1* receptors (44, 45). For example, *CX3CR1*-expressing NK cells and cytotoxic T lymphocyte cells contain perforin and granzyme B, and *CX3CL1*-expressing vascular endothelial cells can effectively activate these immune cells, leading to the release of perforin and granzyme B and ultimately inducing vascular injury (46, 47). In addition, both *CX3CL1* and its receptor *CX3CR1* are expressed in vascular smooth muscle cells and can further attract macrophages to aggregate blood vessels, thereby inducing matrix metalloproteinase (MMP)-mediated extracellular matrix protein hydrolysis as well as promoting smooth muscle cell migration to endothelial cells, ultimately mediating vascular injury (48–50). Notably, the involvement of macrophages, smooth muscle cells, and MMP in the pathological alteration of AA and AA progression has been extensively studied (51–53). It was shown that by inhibiting *CX3CR1*-mediated signaling in smooth muscle cells, the formation of the neointima after arterial injury could be effectively reduced (54). Therefore, *CX3CL1* and its receptor *CX3CR1* cells are present in AA disease, and their interaction contributes to the recruitment and activation of multiple immune inflammatory cells in AA tissue, ultimately promoting AA progression (55). In our study, *CX3CR1* expression was significantly higher in AA tissues than in non-AA tissues, suggesting it could be used as a biomarker for AA. Based on a review of the literature and our previous studies, we have every reason to believe that *CX3CL1* and its receptor *CX3CR1* are potential pharmacological targets for AA treatment.

The *HBB* gene encodes a protein called beta-globin, a subunit of hemoglobin located within red blood cells. Hemoglobin can usually move through the bloodstream and carries oxygen to tissues throughout the body. It has been shown that hypoxic environments may promote hemoglobin-oxygen binding and angiogenesis in Tibetan pigs through transcriptional upregulation of *HBB* expression to adapt to the plateau environment (56). In AAA, *HBB* is considered a potential biomarker in plasma samples from AAA patients (57). Our study substantiated that *HBB* gene expression is higher in AA than in non-AA tissues and may serve as a biomarker for AA. However, it is worth noting that detailed and in-depth studies of the *HBB* gene in AA tissues are still lacking, and it is well-established that *HBB* gene mutations are associated with several severe hemoglobinopathies, such as sickle cell anemia and β-thalassemia (58). We believe that the role of the *HBB* gene in AA warrants further attention, which may facilitate understanding of the molecular mechanisms underlying AA progression and the development of potential drug targets.

In the present study, the microarray expression data of TAA and AAA were merged to obtain common DEGs. We further performed GO, KEGG, DO and GSEA analyses to understand the common biological functions, signaling pathways and disease mechanisms involved in AAA and TAA. GO analysis exhibited significant enrichment in BP, including leukocyte cell-cell adhesion, leukocyte migration, regulation of angiogenesis, regulation of vasculature development and regulation of immune effector process, which is consistent with findings of previous study that chronic inflammation and regulation of angiogenesis play a crucial role in the pathology of AA (34, 35). KEGG analysis showed significant enrichment in Leishmaniasis, Tuberculosis, Th17 cell differentiation, Leukocyte transendothelial migration and cell adhesion molecules. We further performed GSEA analysis to identify the signaling pathways involved in AA and non-AA and found that complement and coagulation cascades, cytokine-cytokine receptor interaction, hematopoietic cell lineage, Leishmania infection, and systemic lupus erythematosus were significantly enriched in AA. The above analyses suggested that inflammatory factor chemotaxis, complement and coagulation cascades, and cell adhesion were commonly involved signaling pathways in AA. An increasing body of evidence suggests that AA is a chronic inflammatory disease involving extensive inflammatory cell infiltration into the arterial wall from the luminal lining to the periaortic epithelium (59, 60), and infiltration of inflammatory factors is closely associated with cytokine chemotaxis and cell adhesion (61, 62). In addition, in our study, complement and coagulation cascades were significantly enriched in AA. The complement system is widely acknowledged to be part of innate immunity. In AA, activation of the innate immune response by auto or foreign antigens further activates the complement system and induces a series of inflammatory cascades in the body, thus promoting disease progression in AA (63, 64). For instance, serum C5a complement levels were significantly elevated in AA patients and correlated with AA diameter (65). In addition, the complement system can be involved in AAA progression by participating in vascular remodeling (66). In the coagulation cascade, fibrinogen and platelet activation play an essential role in the formation of AA (67–69). Anticoagulants, such as low molecular heparin and rivaroxaban, have also been shown to inhibit the progression of AA (70). In summary, these results provide a deeper understanding of the mechanisms of AA progression and provide a basis and direction for further studies in the future.

The broad activation of the immune system in AA has been demonstrated (71, 72), but the mechanisms of co-immune infiltration in TAA and AAA remain largely unclear. Previous researchers have used algorithms such as “CIBERSORT” or “ssGSEA” when assessing the level of immune cell types infiltration in tissues. Such results may not be comprehensive and insightful. However, in our study, these two algorithms, “CIBERSORT” and “ssGSEA” were applied to assess the level of immune infiltration in AA compared to non-AA and biomarkers associated with immune infiltrating cell types. Our results suggest that the innate and adaptive immune systems are heavily activated in AA compared to non-AA, consistent with the literature (73). However, no significant difference in neutrophils, effector memory CD8+ T cells and immature dendritic cells were found between the two groups. Furthermore, resting mast cells positively correlated with resting memory CD4 T cells, while regulatory T cells negatively correlated with resting memory CD4 T cells and resting mast cells. Regulatory T cells (Tregs) are a specific T cell subtype, CD4+Foxp3+, that play an important role in regulating inflammatory responses and maintaining immune homeostasis. Studies have shown that Tregs are involved in protecting against AA through multiple mechanisms (74, 75). Mast cells are another inflammatory cell type found in aortic lesions. The mast cell protease, active chymase, is involved in the activation of MMP, which has been detected in patients positive for this enzyme in AA (76). Besides, mast cells can release pro-inflammatory chemokines involved in the progression of AA (77). Finally, we assessed the correlations between the biomarkers *CX3CR1* and *HBB* and infiltrating immune cells. Indeed, a deeper understanding of this correlation is essential to break the vicious cycle caused by immune-inflammatory activation in AA and identify new therapeutic targets. In our study, *CX3CR1* was highly correlated with macrophage, mast cell, MDSC, dendritic cell, monocytes, Tregs, CD56 bright natural killer cell, central memory CD4 T cell, activated CD8 T cell, gamma delta T cell, T follicular helper cells, and type 1 T helper cell. Moreover, it has a positive correlation with resting mast cells, resting memory CD4 T cells, monocytes, M2 macrophages and gamma delta T cells and a negative correlation with CD8 T cells and Tregs. Besides, *HBB* was highly correlated with eosinophils, mast cells, neutrophils, dendritic cells, monocytes, Tregs, natural killer cells, MDSC, activated CD4 T cells, activated CD8 T cells, effector memory CD4 T cells and T helper cells. It has a positive correlation with activated dendritic cells and eosinophils and a negative correlation with M1 macrophages. Combining the results of both immune infiltration analyses, we found that neither *CX3CR1* nor *HBB* was involved in B cell-induced humoral immunity. Furthermore, a higher correlation was found between *CX3CR1* and macrophages and HBB and eosinophils. In summary, we used two immune infiltration analysis algorithms to adequately assess the level of immune cell types infiltration in AA and to determine the relationship between infiltrating immune cell types and biomarkers. This study provides novel insights into immune mechanisms and designing new immunotherapeutic targets.

Aortic dissection (AD) is usually caused by diseases that can lead to medial degeneration and increased stress on the aortic wall, such as AA and hypertension. AD is also a serious complication of AA. In this process, chronic inflammation, immune activation and stable degradation of extracellular matrix proteins are the key pathological changes that lead to dramatic changes in the aortic wall structure and ultimately to aneurysm formation and AD (1). Therefore, 78 patients with AD were selected for further evaluation of their clinical characteristics, serum inflammation and lipid levels. According to our results, the incidence was higher in men than in women, with a concentration of age of onset in the 50-60 years. Those with a previous history of hypertension had a high incidence and were more likely to be Stanford type B patients with AD. These findings are consistent with the epidemiological studies of aortic disease guidelines and suggest that hypertension is an important causative factor in AD (29, 78). Furthermore, in our study, serum levels of the four lipids were more often in the normal range in patients with AD, indicating that although statins are beneficial for other cardiovascular diseases, they may be less beneficial for patients with AD, especially since the 2018 AAA guidelines clearly stated that they do not reduce the risk of AAA diameter growth (3). In addition, we further assessed the level of inflammation in AA progression to AD at the clinical serum level compared to the previous analysis of immune infiltration at the AA tissue level. We found significantly elevated inflammatory markers (WBC and NEUT%) in patients with AD, which is consistent with previous studies indicating the critical involvement of inflammation in the progression of AD (1). In summary, inflammation and immunity play an important role in aortic disease and are key in studying drugs for treating aortic disease. However, some limitations of this retrospective study should be considered, for instance, the sample size, emphasizing the need for validation in prospective and larger sample studies. In addition, only the inflammatory markers and lipid levels at the time of hospitalization were counted in this study.

It has been established that genetic variants predispose individuals to these thoracic and abdominal aortic diseases: aortic aneurysms (AAA and TAA), aortic dissections (AD), and aortic ruptures (7, 79). Whole-exome sequencing was used to characterize gene mutations in aortic disease and assess whether the screened biomarkers (*CX3CR1* and *HBB)* were mutated. The sequencing of 6 patients with AD showed that the Ti/Tv ratio was close to 2, indicating no large bias in the variant calling process. In addition, the mutated genes screened by the mutation frequency database ESP6500, ExAC and 1000 Genomes were meaningful and valuable. Among the top mutated genes, *ARSD* exhibited the highest number of mutations. Moreover, 100% mutation frequency of *ATN1*, *HRCT1*, *KRT4*, *LNP1*, *MUC4*, *RP1L1* and *TRBV7-6* was observed in 6 samples and 83% for *TTN* and *KDM6B*, while the biomarkers *CX3CR1* and *HBB* were not mutated in the six sequenced samples. Although we identified highly mutated genes in the six samples, few studies have reported mutations in these genes in aortic disease. Moreover, the correlation analysis of 13 mutated genes in 6 samples showed no correlation. Finally, we used DGIdb to make predictions of potentially druggable genes (80), and the results showed that the top 5 genes (*ARSD, ASPN, MUC16, MUC4, TTN)* were highly associated with druggable properties. The development of drugs for aortic diseases from a genetic variant perspective is very promising and may lead to breakthroughs in treating aortic diseases. However, this limitation is similar to our retrospective clinical study in which the sample size of this sequencing was too small, and a prospective study with a larger sample is required to validate our conclusions. In addition, the characteristics of the population, environmental factors and differences in genetic susceptibility may impact the conclusions.

## Conclusion

Overall, we innovatively merged the TAA and AAA data sets to identify two biomarkers (*CX3CR1* and *HBB*) based on two machine-learning algorithms and ROC curves. Furthermore, we conducted different bioinformatics analyses, including GO, KEGG, DO, GSEA and two immuno-infiltration analysis algorithms (“CIBERSORT” and “ssGSEA”). This study demonstrated the common molecular mechanisms and immune infiltration patterns associated with TAA and AAA, the correlations between the biomarkers and infiltrating immune cells, and provided insights into the underlying development of potential immunotherapeutic drugs. Finally, a retrospective clinical study revealed the presence of a hyper-inflammatory environment in aortic disease. The WES study identified mutated genes, biomarkers, and druggable variants genes. Due to the study limitations, further validation in prospective and larger sample studies is warranted for a deeper understanding of the pathological process, immune infiltration mechanisms, targeted drug development and variant genes in aortic disease.

## Data availability statement

The WES data presented in the study are deposited in the NCBI SRA repository (https://www.ncbi.nlm.nih.gov/sra/), accession numbers SRR21846217, SRR21846218, SRR21846219, SRR21846220, SRR21846221 and SRR21846222.

## Ethics statement

The studies involving human participants were reviewed and approved by the Ethics Committee of Affiliated Hospital of Youjiang Medical University for Nationalities (YYFY-LL-2016-06). The patients/participants provided their written informed consent to participate in this study.

## Author contributions

BH was engaged to write and conceptualize the original draft. BH and YZ were responsible for methodology. CC, DY, QW, LQ, DH, YL, and ZL were responsible for software. LL and XP were responsible for reviewing and editing. All authors contributed to the article and approved the submitted version.

## Funding

This study was sponsored by funds from the Chinese National Natural Science Foundation of China (81560076), Guangxi Natural Science Foundation Youth Program(2018JJB140358), Middle-Aged and Young Teachers in Colleges and Universities in Guangxi Basic Ability Promotion Project (2021KY0534), the First Batch of High-level Talent Scientific Research Projects of the Affiliated Hospital of Youjiang Medical University for Nationalities in 2019(R20196316).

## Acknowledgments

It was the GEO network and the Affiliated Hospital of Youjiang Medical University for Nationalities that helped make this study possible. We are very grateful to the patients hospitalized at the Affiliated Hospital of Youjiang Medical University for Nationalities for agreeing to our study. We are also very grateful to the GEO database for providing free data. In additional, we have asked the researcher’s house for help with the language. Thanks to FreeScience (https://www.home-for-researchers.com) for helping with the English language.

## Conflict of interest

The authors declare that the research was conducted in the absence of any commercial or financial relationships that could be construed as a potential conflict of interest.

## Publisher’s note

All claims expressed in this article are solely those of the authors and do not necessarily represent those of their affiliated organizations, or those of the publisher, the editors and the reviewers. Any product that may be evaluated in this article, or claim that may be made by its manufacturer, is not guaranteed or endorsed by the publisher.
